# Interoception, personality, and internet use: Preliminary insights into their association

**DOI:** 10.1371/journal.pone.0328260

**Published:** 2025-07-15

**Authors:** Dovile Simkute, Grace Y. Wang, Inga Griskova-Bulanova

**Affiliations:** 1 Institute of Biosciences, Life Sciences Centre, Vilnius University, Vilnius, Lithuania; 2 School of Psychology and Wellbeing, University of Southern Queensland, Toowoomba, Australia; 3 Centre of Health Research, University of Southern Queensland, Toowoomba, Australia; 4 Faculty of Medicine, Translational Health Research Institute, Vilnius University, Vilnius, Lithuania; University of Belgrade Faculty of Philosophy: Univerzitet u Beogradu Filozofski Fakultet, SERBIA

## Abstract

Problematic Internet Use (PIU) is increasingly recognized as a concern among internet users, prompting investigations into the factors that may predispose individuals to PIU. Interoceptive awareness, one of cognitive-perceptual factors, refers to the ability to perceive internal bodily sensations and has been shown to play a significant role in the onset and maintenance of drug addiction. However, its relationship with PIU remains underexplored. This study aimed to investigate the dimensions of interoceptive awareness within the context of PIU and to explore the role of personality traits in their relationships in a non-clinical sample of regular internet users. Involving 161 participants (71 males), the PIUQ-9 (Nine-Item Problematic Internet Use Questionnaire), DPIU (Dimensions of Problematic Internet Use), Neo-Pi-R NEO (Personality Inventory-Revised) and MAIA (Multidimensional Assessment of Interoceptive Awareness) questionnaires were employed. Spearman correlations and network analysis were conducted to assess relationships and interconnections among the variables. Neuroticism emerged as a central factor, strongly linked to both PIU and interoceptive awareness. Network analysis highlighted specific negative connections between the interoceptive states of Trusting and Not-Distracting and PIU. These preliminary findings suggest that certain interoceptive dimensions and personality traits, particularly neuroticism, are significantly associated with PIU. This study contributes to the field by highlighting interoceptive dimensions as relevant factors in understanding PIU and emphasizes the scarcity of research in this area, encouraging further investigation to address this gap.

## Introduction

Currently, approximately two-thirds of the world population are internet users, a figure that is expected to continue increasing steadily [1]. The widespread use of the internet has profoundly transformed daily work and lifestyles by reducing information disparities, facilitating the dissemination of knowledge, and enhancing access to diverse forms of entertainment. However, alongside these benefits, the growing prevalence of internet use and the consequent rise in Problematic Internet Use (PIU) have prompted the World Health Organization to classify PIU as a global public health concern [2]. Research indicates that excessive and detrimental use of digital media is linked to physical, psychological, social, and neurological adverse effects [3–5]. As Kuss & Lopez-Fernandez stated in their systematic review of clinical research [6], comorbidities are more common among individuals exhibiting signs of PIU, such as depression, anxiety disorders, ADHD, insomnia, and substance use disorders [7–10]. Although mental health issues are often associated with PIU, psychopathology alone is not a sufficient factor for the development of the disorder [11].

Interoceptive awareness, a key cognitive-perceptual factor, refers to the ability to perceive internal bodily sensations. It has been identified as playing a significant role in addiction through its influence on various psychological processes, such as response to stress, emotional regulation, self-esteem, and mental health conditions, including impulsivity, loneliness, depression, and anxiety [12,13]. Misinterpretations of bodily signals may exacerbate these psychological states, predisposing individuals to addictive behaviors [14]. For instance, inadequate interoceptive processing can lead to dysfunctions in approach and avoidance behaviors related to addictive substances, with interoceptive awareness influencing decisions based on the body’s need to maintain homeostasis [15]. Additionally, interoception impacts emotional processing, decision-making, and craving processes, balancing the interplay between impulsive and reflective systems [16–20]. While the role of interoceptive awareness is well-established in the onset and maintenance of drug addiction, few research has examined its impact in the context of behavioral addiction.

A recent survey by Di Carlo et al. [21] revealed that individuals at risk for PIU exhibited reduced interoceptive abilities in perceiving bodily sensations (the “noticing” dimension), a heightened tendency to deny bodily sensations (the “not-distracting” dimension), and an excessive propensity to worry about negative bodily signals (the “not-worrying” dimension) compared to individuals not at risk. Interestingly, the study found that the severity and impact of PIU may be influenced not only by individual interoceptive sensitivities but also by the interactions between these dimensions. Consistent with this, structural and functional changes in the insular cortex, a key region involved in interoception, have been identified in individuals with Internet Gaming Disorder (IGD) [22–26]. It is proposed that interoceptive accuracy is an integral component of the triadic neurocognitive model of addiction [27] and is closely connected to individuals’ psychological states, by generating a craving state through the transformation of bodily signals into a subjective drive, thus contributing to addiction.

Furthermore, how individuals interpret internal biological signals can be influenced by personality traits. Individuals with high extraversion tend to focus on positive aspects, while those with higher levels of neuroticism are more attuned to negative aspects [28]. Research suggests that individuals with specific attributional styles for life events may also apply these styles to their interoceptive perceptions [28]. Moreover, internet behaviors are not a homogeneous construct and pattern of use and associated consequence could be influenced by many factors. The broad categorization of all problematic online behaviors in to one umbrella term PIU could be problematic [29,30], failing to account for the heterogeneity of problem behaviors [30]. The choice of specific internet activities is significantly shaped by the motivations for engagement – factors intertwined with both psychopathology and personality traits [31]. For example, certain online behaviors, such as social media use, are more closely associated with the motives of social interaction, self-enhancement, and information-seeking [32,33]. In contrast, activities like gaming are often linked to motivations such as sensation-seeking, emotional escapism, a desire for competition, or reward-seeking [34,35]. Disaggregating PIU by the type of platform or activity could help to identify specific factors associated with each behavior, contributing to a more nuanced understanding and more targeted and effective strategies for managing PIU.

While interoceptive awareness has been widely studied in the context of substance-related addictions, its role in behavioral addictions such as PIU has received limited attention (to date, we have identified only one study [21] directly addressing this topic). This gap highlights the need for empirical research examining how (if) interoceptive processes may influence or reflect patterns of problematic internet engagement. The current study aims to examine the dimensions of interoceptive awareness in relation to the risk of PIU within a non-clinical sample and to explore their associations with personality traits and specific types of internet use. Based on existing literature (i.e., [21,31]), we hypothesize an inverse relationship between interoceptive awareness and the severity of PIU. Specifically, lower interoceptive awareness is expected to be associated with higher levels of PIU [21]. We also expect that personality traits, particularly higher neuroticism, would be positively associated with greater internet use and negatively associated with interoceptive awareness, given its link to emotional reactivity and regulation difficulties [31,36–39]. Additionally, we aim to explore whether distinct interoceptive dimensions are differentially associated to specific domains of internet use behavior. Findings of this research would help to reveal the link between individuals’ self-awareness of bodily states and patterns of internet engagement, thereby expanding the understanding of psychological factors that could serve as early warning signs for PIU.

## Methods

### Participants

The sample consisted of 161 participants (71 males) with a mean age of 24.21 years (SD = 4.28 years). Exclusion criteria for adult participants included a history or current diagnosis of psychiatric, neurological, or endocrinological disorders, use of psychotropic or psychoactive substances, and self-considered or clinically confirmed addictions. Recruitment was carried out through advertisements in the student community, social media, and various media platforms. The study was conducted in a Lithuanian sample and was part of a larger experimental project investigating the neural correlates of internet usage patterns in a non-clinical sample of self-considered regular internet users. Participation was voluntary, with written informed consent obtained; the study was approved by the Regional Biomedical Research Ethics Committee (Nr.2019/10-1159-649), and all participants gave their written informed consent.

### Measures

The self-report measures analyzed in the present manuscript (DPIU, PIUQ-9, MAIA, NEO-PI-R; described below) were administered after EEG-related tasks, which lasted approximately 30 minutes.

**The Multidimensional Assessment of Interoceptive Awareness** (**MAIA**), developed by Mehling et al. [40], is a self-report questionnaire designed to assess an individual’s interoceptive awareness, which is the ability to perceive and interpret bodily sensations. The MAIA consists of 32 items, each rated on a six-point Likert scale ranging from 0 (never) to 5 (always). The MAIA is divided into eight subscales, each measuring a different dimension of interoceptive awareness: Noticing (the ability to consciously perceive uncomfortable, comfortable, or neutral bodily sensations), Not-Distracting (the tendency to remain present with discomfort rather than avoiding it through distraction), Not-Worrying (the capacity to experience bodily sensations without significant emotional distress), Attention regulation (the ability to intentionally focus on and control attention to bodily sensations), Emotional awareness (the ability to recognize the connection between physical sensations and emotions), Self-regulation (the capacity to utilize awareness of bodily sensations to regulate emotions), Body listening (the tendency to actively listen to the body for insights), and Trusting (the extent to which one views their body as a safe and trustworthy). The MAIA is a valuable tool for research and clinical practice, providing a comprehensive assessment of interoceptive awareness [40]. The scale has been validated in a Lithuanian sample [41,42]. The Cronbach’s alpha values for the present study were as follows: whole scale (α = 0.88), subscales: noticing (α = 0.67), not distracting (α = 0.58), not worrying (α = 0.55), attention regulation (α = 0.83), emotional awareness (α = 0.81), self-regulation (α = 0.8), body listening (α = 0.82) and trust (α = 0.87). Consistent with other authors, we found two subscales showing less optimal values; however, evidence supports its validity and discriminant ability in use [43,44]. While it should be noted that a revised version of the instrument (MAIA-2) has recently been developed to address psychometric limitations of the original version, it has not yet been validated in the Lithuanian language. Therefore, the original version was used in the present study.

**The Nine-Item Problematic Internet Use Questionnaire (PIUQ-9)** [45] is a short 9-item questionnaire designed to measure problematic internet use. Scores range from 9 to 45, with a provisional cutoff of 22 indicating problematic use. The scale has been validated in a Lithuanian sample [46], and in the current sample it has a Cronbach’s alpha of 0.81.

**The Dimensions of Problematic Internet Use** (**DPIU**) [47] is a self-report tool designed to assess problematic internet use based on DSM-IV criteria for addiction [48] and covers nine different dimensions of PIU: Entertainment and Video Streaming, Social Media, Gaming, Messaging, Dating Apps, Gambling, Sexual Content, Online Shopping, and Information Search. Every dimension starts with three initial screener questions (YES/NO). For instance, in the Entertainment and Video Streaming dimension, these questions include: “Have you ever felt bad or guilty about how much you spend streaming?”, “Have people ever annoyed you by criticizing how much time you spend streaming?”, and “Have you ever felt you should cut down on the amount of time you spend streaming videos?”. If respondents answer “yes” to two or more questions, they proceed to five additional questions on a 6-point Likert scale ranging from 0 (not at all) to 5 (a lot) assessing potential problematic usage within that dimension. Individuals with scores 3 or greater in any of the dimensions indicate a potential risk of PIU. The number of participants reporting problematic use in each dimension and Cronbach’s alpha values were as follows: Entertainment and Video Streaming: n = 103, α = 0.8, Social Media: n = 99, α = 0.77, Gaming: n = 37, α = 0.86, Messaging: n = 43, α = 0.84, Dating Apps: n = 12, α = 0.68, Sexual Content: n = 16, α = 0.68, Online Shopping: n = 21, α = 0.82, Information Search: n = 25, α = 0.74. There was no participant reporting problematic use in Gambling, n = 0.

**The NEO Personality Inventory-Revised** (**NEO-PI-R**) [49] in its 240-item version, is a comprehensive self-report questionnaire designed to assess an individual’s personality based on the Five-Factor Model (FFM). This model encompasses five broad domains of personality: Neuroticism, Extraversion, Openness to Experience, Agreeableness, and Conscientiousness. Each item on the NEO-PI-R is rated on a five-point Likert scale ranging from “strongly disagree” to “strongly agree. Respondents are asked to indicate the extent to which they agree or disagree with various statements about their thoughts, feelings, and behaviors. The 240 items provide a detailed assessment of each of the five domains and their corresponding facets, allowing for a nuanced understanding of an individual’s personality profile. The NEO-PI-R has been translated into numerous languages and is used globally to assess personality traits in diverse populations. Cronbach’s alpha of the factors indicated a high degree of internal consistency: Neuroticism α = 0.92, Openness to Experience α = 0.9, Extraversion α = 0.89, Agreeableness α = 0.89, Conscientiousness α = 0.91.

### Data analysis

A multi-method approach was employed to examine the relationships among study variables. Correlations were used to assess the relationship between interoceptive awareness, the risk of PIU and personality traits. Linear regression was conducted to evaluate the independent contribution of interoceptive dimensions and personality traits to problematic internet use, and network analysis was applied to model the multivariate structure of these relationships. This combination of methods allowed for the investigation of both specific associations and broader interaction patterns within the dataset.

The Kolmogorov-Smirnov and Shapiro-Wilk tests were conducted to test for normality, revealing that not all variables were normally distributed and necessitating the use of non-parametric tests. Since 87% (140/161) of participants fully completed all the questionnaires and the percentage of missing data in the questionnaires ranged from 0 to no more than 6.2%, the pairwise exclusion method was selected to manage the missing data [50].

Spearman correlations were performed to examine the relationships between interoception (MAIA), personality traits (NEO-PI-R), and internet use (PIUQ-9, DPIU). A linear regression model was subsequently employed to evaluate the predictive value of interoceptive awareness (MAIA) and personality traits (NEO-PI-R) for the risk of problematic internet use (PIUQ-9); the normality of the standardized residuals for the linear regression model was assessed visually using a Q-Q plot, which indicated approximate normality. Additionally, network analysis was conducted to explore the dynamic interactions between interoceptive dimensions, personality factors, and internet use patterns. The extended Bayesian information criterion (EBIC) alongside a hyperparameter set at 0.5 and non-parametric bootstrapping (with 1000 bootstrap samples) was used to ensure a balance between specificity, interpretability, and sensitivity [51]. The common centrality measures (strength, closeness, betweenness, and expected influence) [52] were evaluated to assess the importance and connectivity of variables within the network. The results are visualized as a weighted network structure, where each node represents a variable, and each edge represents a correlation, with blue edges indicating positive and red edges indicating negative relationships. Edge thickness and color saturation reflect the strength of these correlations. Data were analysed using IBM SPSS statistics (Version 29) for the descriptive and inferential tests and JASP software [53] for the network analysis.

Statistical power was evaluated using a *post hoc* power analysis with G*Power (version 3.1.9.4; [54]). For bivariate correlations, a sample size of 161 provided 80% power to detect correlations of approximately r = 0.22 (two-tailed, α = .05), representing small-to-moderate effect sizes. For the multiple linear regression analysis with nine predictors, this sample provided 94% power to detect an overall model effect size of f^2^ = 0.15. While formal sample size guidelines for the network analysis are still evolving, recommendations suggest that larger samples contribute to more accurate and stable parameter estimation [51].

## Results

Descriptive statistics (means and standard deviations) for the Internet Usage patterns, Interoceptive Awareness, and Personality Traits assessed in the study sample are presented in Table 1. Due to the small number of participants reporting usage of the Dating Apps (n = 12), Sexual Content (n = 16) and Online Shopping categories (n = 21), these domains were excluded from the following analysis.

**Table 1:** Descriptive statistics of internet usage patterns, interoceptive awareness, and personality traits in the sample.

Variable	Mean	SD
**Internet Usage Patterns (PIUQ-9, DPIU)**		
PIUQ-9	19.68	5.43
DPIU_total	25.45	18
Entertainment and Video Streaming	9.84	4.49
Social Media	9.26	4.33
Gaming	11.97	5.32
Messaging	12.77	5.35
Dating Apps	7.75	3.98
Sexual Content	10.25	3.99
Online Shopping	9.05	4.73
Information Search	12.56	4.42
**Interoceptive Awareness (MAIA)**		
Noticing	3.34	0.93
Not-Distracting	2.97	0.91
Not-Worrying	3.02	1.10
Attention Regulation	3.00	0.83
Emotional Awareness	3.28	1.05
Self-Regulation	2.71	1.09
Body Listening	2.26	1.17
Trusting	3.64	1.02
**Personality Traits (Neo-Pi-R)**		
Neuroticism	52.63	12
Extraversion	48.58	11.14
Openness	58.00	10.35
Agreeableness	48.98	12.15
Conscientiousness	49.87	10.79

Note. DPIU, dimensions of problematic internet use; MAIA, multidimensional assessment of interoceptive awareness; NEO-PI-R, revised NEO personality inventory; PIUQ-9, problematic internet use questionnaire.

### Relationships between interoceptive awareness and internet use

Significant relationships were observed between interoceptive awareness and PIU (Table 2). The risk of problematic internet use indicated the PIUQ-9 scores was significantly negative correlated with Not-Distracting (r_s_ = −.36, p < 0.001). Regarding the dimensions of internet use, the Information Search domain exhibited a significant positive correlation with Not-Worrying (r_s_ = .44, p = 0.03), while Messaging domain demonstrated significant negative correlations with Attention Regulation (r_s_ = −.4, p = 0.008), Emotional Awareness (r_s_ = −.4, p = 0.01), Self-Regulation (r_s_ = −.46, p = 0.003), Body Listening (r_s_ = −.47, p = 0.002) and Trusting (r_s_ = −.31, p = 0.05). No significant correlations were observed between interoceptive domains and Entertainment and Video Streaming, Social Media, or Gaming dimensions. Results are presented in Table 2 and full correlational matrix is provided in S1 Table in S1 File.

**Table 2:** Spearman’s correlations between interoceptive awareness and personality traits and internet usage patterns.

Variable	Noticing	Not-Distracting	Not-Worrying	Attention Regulation	Emotional Awareness	Self-Regulation	Body Listening	Trusting
**Personality Traits (Neo-Pi-R)**								
Neuroticism	−0.06	−0.15	−0.39*	−0.37*	0.17*	**−0.39**	−0.11	−0.45*
Extraversion	0.05	0.08	0.08	0.06	0.1*9	0.17*	0.1*9	0.2*
Openness	0.2*	0.08	−0.05	0.13	0.28*	0.18*	0.2*8	0.05
Agreeableness	−0.03	0.1	−0.07	−0.02	0.07	0.08	0.11	0.08
Conscientiousness	0.08	0.06	−0.14	0.15	0.04	0.14	0.12	0.21*
**Internet Usage Patterns (PIUQ-9, DPIU)**								
PIUQ-9	0.03	−0.36*	−0.08	0.06	0.1	−0.04	0.06	−0.16*
DPIU_total	0.08	−0.2*	−0.004	−0.11	0.07	−0.08	0.01	−0.17
Entertainment and Video Streaming	0.09	−0.19	−0.08	−0.15	−0.007	−0.02	0.01	−0.16
Social Media	−0.08	−0.04	−0.07	−0.07	0.06	0.01	0.01	−0.03
Gaming	0.16	−0.09	−0.23	0.12	0.24	0.28	0.31	0.12
Messaging	−0.1	0.02	−0.08	−0.4*	−0.4*	−0.46*	−0.47*	−0.31*
Information Search	−0.13	−0.23	0.44*	−0.11	−0.18	−0.05	−0.34	0.08

Note. Significant correlations are marked by asterisks (*). DPIU, dimensions of problematic internet use; MAIA, multidimensional assessment of interoceptive awareness; NEO-PI-R, revised NEO personality inventory; PIUQ-9, problematic internet use questionnaire.

### Association between interoceptive awareness and personality traits

Significant relationships were observed between interoceptive awareness and personality traits across all traits, except for Agreeableness (Table 2). After applying an alpha correction to account for multiple comparisons (p = 0.05/40 = 0.001), personality traits significantly correlated with interoceptive awareness were Openness and Neuroticism. Specifically, Openness was positively correlated with body listening (r_s_ = .28, p < 0.001) and emotional awareness (r_s_ = .28, p < 0.001), while Neuroticism was negatively correlated with self-regulation (r_s_ = −.39, p < 0.001), attention regulation (r_s_ = −.37, p < 0.001), not-worrying (r_s_ = −.39, p < 0.001) and trusting (r_s_ = −.45, p < 0.001).

### Contribution of different interoceptive variables and neuroticism to PIU

**Fig 1:**
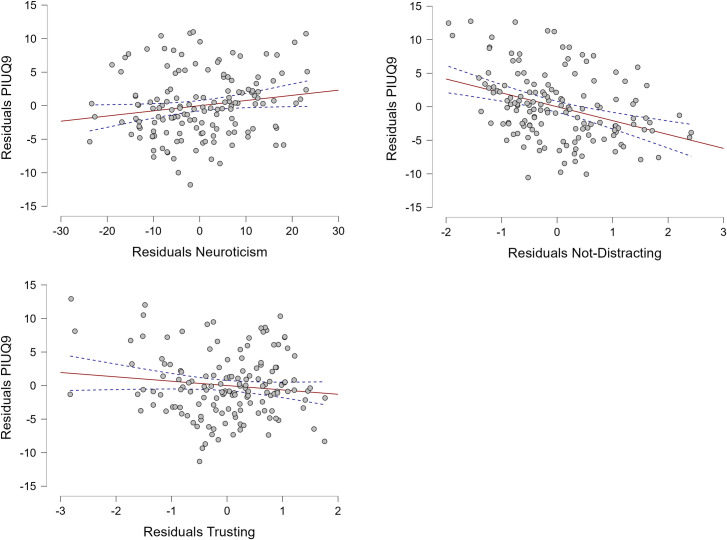
Partial regression plots of statistically significant predictors of Problematic Internet Use.

In the linear regression analysis three variables – Neuroticism (p = 0.04), Not-Distracting (p < 0.001), and Trusting (p = 0.04) – were found to be statistically significant predictors of PIU (Fig 1). The model achieved R value of 0.509, indicating a moderate positive linear relationship between the predictors and PIU, and explained 25.9% of variability in PIU score. Results are presented in Table 3.

**Table 3:** Linear regression model assessing the independent contribution of neuroticism and different interoceptive awareness variables to PIUQ-9 score.

Predictors	*B*	*SE*	*β*	*t*	*p*	*95% CI*
(Intercept)	20.63	4.28		4.82	**< .001**	12.17 to 29.09
Neuroticism	0.09	0.04	0.2	2.08	**0.04**	0.004 to 0.17
Noticing	−0.44	0.53	−0.07	−0.83	0.41	−1.47 to 0.61
Not-Distracting	−1.85	0.46	−0.32	−4.04	**< .001**	−2.75 to −0.95
Not-Worrying	0.18	0.44	0.04	0.42	0.68	−0.68 to 1.04
Attention Regulation	1.16	0.66	0.18	1.76	0.08	−0.143 to 2.46
Emotional Awareness	0.42	0.6	0.08	0.71	0.48	−0.76 to 1.6
Self-Regulation	−0.006	0.53	−0.001	−0.01	0.99	−1.06 to 1.05
Body Listening	−0.16	0.55	−0.04	−0.3	0.77	−1.25 to 0.92
Trusting	−0.99	0.48	−0.19	−2.08	**0.04**	−1.94 to −0.05

Note. Significant values are bolded. **B** = Unstandardized regression coefficient, **SE** = Standard error of B, **β** = Standardized regression coefficient, **t** = t-value for the predictor, **p** = p-value (significance level).

### The network visualization of interoception, personality traits, and PIU

**Fig 2:**
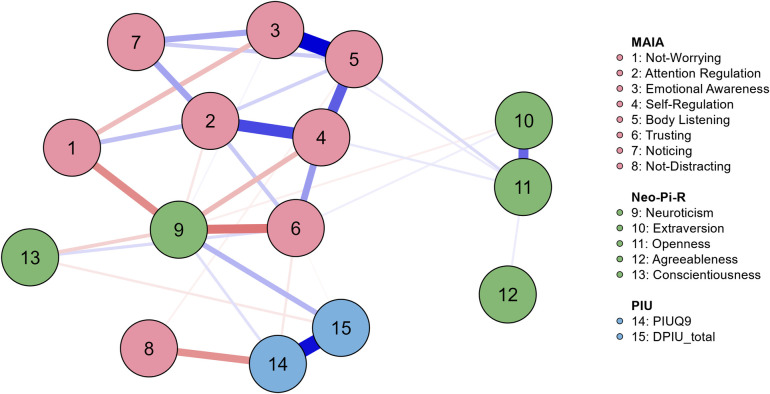
Network of Interoceptive awareness, Personality traits, and Problematic Internet Use network. This diagram includes 15 distinct variables, labeled on the right, with connections depicted through EBICglasso partial correlations. Positive relationships are shown with blue edges, and negative ones with red edges. The width and saturation of these edges indicate the strength of the relationships.

To explore the interrelationships among various interoceptive states, personality traits, and PIU, the EBICglasso method based on covariance correlation coefficients with a tuning parameter of 0.5, using 1000 non-parametric bootstrapped samples was employed to construct a network of eight interoception state variables, five personality traits, and two PIU measures (Fig. 2). Out of 105 potential edge weights, 33 were non-zero, resulting in a network sparsity of 0.686. The weighted edge matrix is detailed in S2 Table in S1 File.

The network analysis showed Neuroticism as a key intermediary in the network, acting as a “bridge” between PIU and interoceptive awareness variables. As anticipated, both the PIUQ-9 and DPIU questionnaires showed connections to Neuroticism (0.053, 0.129, respectively). Within interoceptive states, Neuroticism was negatively associated with Trusting (−0.239) and Not-Worrying (−0.202) variables, along with weaker connection to Self-Regulation (−0.124).

The analysis revealed that both PIU questionnaires (PIUQ-9 and DPIU) showed connections to each other (0.421), but neither was strongly connected to most interoception dimensions, except for negative links from PIUQ-9 to Not-Distracting (−0.192).

**Fig 3:**
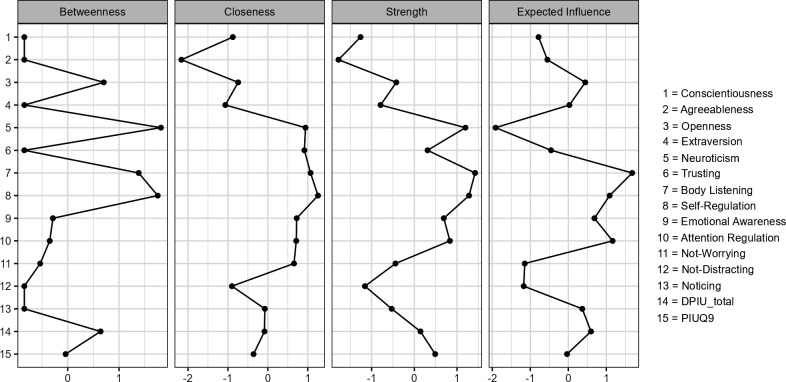
Centrality Indices for the Network of Interoceptive Awareness, Personality Traits, and Problematic Internet Use. The horizontal axis shows standardized (Z) centrality scores, while the vertical axis lists each variable in the network. Variables exhibiting higher centrality are positioned further to the right, away from the vertical axis.

The standardized centrality indices for betweenness, closeness, degree, and expected influence are presented in Fig. 3. The degree index highlights Neuroticism, Body Listening, and Self-Regulation as the nodes with the most connections. In terms of betweenness, Neuroticism, Self-Regulation, and Body Listening are positioned as the nodes with the shortest pathways connecting other variables in the network. Considering closeness, the index shows Self-Regulation as having the highest inverse sum of shortest paths to all other nodes in the network. For expected influence, the nodes with the highest cumulative edge weights, considering both positive and negative relationships, are Body Listening, Self-Regulation, and Attention Regulation. The precision of these edge weights was assessed using 95% bootstrapped confidence intervals, with an illustration of the estimated edge-weight matrix and these intervals presented in S1 Fig in S1 File.

## Discussion

The present study aimed to investigate the link between dimensions of interoceptive awareness and risk of PIU in a non-clinical sample and to explore its associations with personality traits and specific types of internet use. However, as this study is based on a relatively small sample, the results should be considered preliminary and interpreted with caution, serving as a basis for future confirmatory research.

Awareness of bodily sensations, such as pain, arousal, and body temperature, influences cognitive-affective effects associated with drug use and shapes subjective bodily feedback, enabling flexible and rapid responses [55]. Research shows that patients with heightened interoceptive awareness are more likely to experience intense drug urges and face an elevated risk of relapse triggered by negative mood or stress. Decreased interoception awareness has been observed in patients addicted to alcohol, heroin, or synthetic cannabinoids [56]. In the context of PIU, where no chemical substances are involved, we found that the risk of PIU was significantly negatively correlated with the Not-Distracting dimension of interoceptive awareness. Specifically, individuals with a lower tendency to avoid or distract themselves from pain or discomfort exhibited a greater risk of PIU. Similarly, those with lower subjective trust in their perceived bodily states were at higher risk of PIU. Evidence suggests that when a person has poor interoceptive awareness, they may be unable to effectively utilize bodily signals. Instead, they might rely on non-emotional sources to guide their decision-making, and this can lead to choosing more disadvantageous and fewer advantageous options in complex and uncertain decision-making situations [57]. It appears that diminished interoceptive awareness may impair the ability to self-regulate and assess risks effectively, which could explain the susceptibility to problematic internet use.

Furthermore, neuroticism emerged as a central factor in the network of personality traits, interoceptive states and internet use patterns, linked to either risk of PIU or impaired interoceptive awareness. Neuroticism has been identified as a key personality trait linked to PIU, as evidenced by numerous studies, including meta-analyses and reviews [58–60]. As suggested by Lam et al. [38] in a systematic review on risk factors for internet addiction, lower self-control and neuroticism (among higher impulsivity, sensation-seeking, and social inactivity), may disproportionately alter the balance of daily activities and lead to online engagement. The meta-analysis by Marciano et al. [31] which was based on 159 studies on PIU and neuroticism, illustrate maladaptive engagement to online environments as behaviors, often stemming from pre-existing psychological vulnerabilities and emphasize neuroticism’s interplay between personality characteristics (such as heightened sensitivity to negative emotions, emotional instability, poor self-regulation, among others), dysfunctional coping strategies, and environmental stimuli, rather than being a direct consequence of problematic usage of internet related activities. As such, neuroticism (not as independent trait, but as expression of underlying dysfunction) predisposes individuals to problematic online behaviors by amplifying pre-existing psychological traits and vulnerabilities (such as emotional and interpersonal difficulties), creating ground for maladaptive coping in response to environmental triggers (like rewarding online experiences in various internet platforms).

In support of the above, negative connections between neuroticism and interoceptive domains such as Not-Worrying, Self-Regulation, and Trusting were observed in the current study. These results are in line with a previous study by Gaggero et al. [39], which also showed that subjects high in neuroticism have interoceptive deficits across the same domains. The observed associations suggest that individuals with higher neuroticism may face difficulties in perceiving and responding effectively to bodily sensations. Specifically, this includes heightened emotional distress to discomforting sensations (Not-Worrying dimension), challenges in regulating psychological distress to these states (Self-Regulation dimensions), and a decreased ability to perceive one’s body as reliable or safe (Trusting dimension) [40]. Such results align with suggestions that neuroticism may predispose individuals to engage in digital activities as a coping mechanism, associated with lower self-regulation abilities [36,37]. In this context, neuroticism emerges as both a predisposing marker and a potential therapeutic target in addressing maladaptive PIU patterns, particularly through interventions aimed at enhancing emotional regulation, such as mindfulness-based or cognitive-behavioral approaches [61,62].

To our knowledge, only one study investigated PIU within a context of interoceptive awareness [21]. Di Carlo et al. [21] research highlighted significant differences between the PIU (measured by IAT) and non-PIU groups, particularly in the domains of Not-Distracting, Trusting, Not-Worrying, and Emotional Awareness. While our results confirm the involvement of Not-Distracting and Trusting dimensions in PIU, similar to as found by Di Carlo et al. [21], the associations with Not-Worrying and Emotional Awareness domains in the current study appeared to be more domain-specific rather than general. Specifically, Messaging domain exhibited negative correlations with the most interoceptive variables, including Attention Regulation, Self-Regulation, Body Listening, Emotional Awareness, and Trusting. In contrast, Information Search domain was specifically associated with Not-Worrying variable only. Meanwhile, the remaining domains – Entertainment and Video Streaming, Social Media, and Gaming – showed no significant correlations. This contrast may be partly attributed to differences in the conceptualization and measurement of PIU: while Di Carlo et al. [21] focused on general PIU risk using a single IAT threshold to define PIU and non-PIU groups, the current study employed a multi-dimensional approach (PIUQ-9 and DPIU), assessing PIU as a continuous construct across the full sample and aiming to capture variation across specific internet activities. Additionally, the current findings suggest that emotionally and cognitively demanding activities, such as messaging [63,64], may interact more directly with bodily self-regulation mechanisms, indicating that certain interoceptive processes could be more sensitive to contextual internet use rather than global usage patterns. These findings highlight the diverse ways in which different internet activities might engage with or dissociate from internal bodily awareness and emphasize the complexity of interoceptive dimensions in relation to PIU, highlighting both shared and distinct patterns across studies. Moreover, Di Carlo et al. [21] examined alexithymia and found that participants with higher internet engagement exhibited elevated levels of alexithymia – particularly difficulties in identifying emotions and a tendency to focus on external stimuli rather than internal emotional states – suggesting a reliance on the internet as a form of emotional regulation, likely in a bidirectional manner. In contrast, and as discussed above, the current study focused on personality traits, suggesting an alternative but complementary pathway in which neuroticism may be also associated with disrupted bodily awareness and problematic internet use.

### Limitations

The relatively small sample size (n = 161) of this study limits the generalizability of the findings. The statistical power required for detecting subtle effects was limited being particularly evident in the DPIU subdimensions, where the number of participants reporting specific behaviors was small, rendering the data unsuitable for network analysis. Thus, caution should be exercised even when interpreting some correlations. We suggest considering the results of this study as preliminary research, aimed at encouraging other researchers to investigate diverse behaviors within online activities further.

To our knowledge, DPIU, which was constructed based on DSM-5 diagnostic criteria for addictive disorders [47], has not been validated in previous studies. However, both PIUQ-9 and DPIU were selected for this study because of their complementary strengths in assessing problematic internet use. The validated and widely used PIUQ-9 offers a quick and comprehensive assessment of general problematic internet use, capturing individuals’ overall Internet use patterns, while DPIU provides a deeper look into the specific aspects of internet use, categorizing problematic internet use into specific domains like Social Networking, Gaming or Messaging, among others. Together, these tools address both broad and specific dimensions of PIU, providing a comprehensive framework for understanding and addressing problematic internet behaviors. Additionally, they allow the association of each dimension with unique psychological profiles, necessitating a differentiated approach to the study. However, due to the lack of DPIU validation, these results should be interpreted cautiously.

This study has a dual aspect of both a limitation and a strength due to its sampling strategy, which includes healthy individuals who are not clinically diagnosed or self-identified with problematic internet use. The increasing number of Internet users worldwide (Statista) has been a significant factor in the modern era, making it important to study not only problematic behaviors but also casual Internet use. This approach prevents the pathologization of behaviors common among the general population and aids in identifying risk factors that could lead to problematic usage.

## Conclusions

This study provides valuable insights into the intricate interplay between interoceptive awareness, personality traits, and PIU. By employing a network analysis approach, we identified neuroticism as a central personality factor, influencing both interoceptive dimensions and PIU, while interoceptive traits such as Trusting and Not-Distracting emerged as key variables in the context of PIU. The findings underscore the importance of examining PIU as a multifaceted construct, emphasizing the diverse interactions between personality, interoceptive states, and distinct internet domains. This study contributes to the field by highlighting interoceptive dimensions as relevant factors in understanding PIU and emphasizes the scarcity of research in this area, encouraging further investigation to address this gap. Moreover, the current findings may have practical implications for the early identification of individuals who do not meet clinical diagnostic criteria but are nonetheless at risk for developing PIU. Screening for interoceptive deficits in conjunction with elevated levels of neuroticism may help identify these at-risk individuals. Interventions aimed at improving body awareness and emotional self-regulation could be effectively integrated into prevention and mental health programs targeting the risk of PIU.

## Supporting information

S1 File **S1 Table. Spearman’s correlations between interoceptive awareness and personality traits and internet usage patterns. S2 Table. MAIA, Neo-Pi-R and PIUQ-9, DPIU edge weights obtained in the network analysis matrix.S1 Fig. The accuracy and stability of the network for interoceptive awareness, personality traits, and problematic internet use illustrated through bootstrapped confidence intervals (CIs) of estimated edge weights.** In the figure, the x-axis represents the estimated edge-weight coefficients, while the y-axis lists each estimated edge weight in descending order from the highest to the lowest mean bootstrap edge-weight. The red line denotes the sample values, and the black line indicates the mean bootstrapped estimated edge-weights. Larger CIs, depicted by a wider shaded area around the mean bootstrapped estimated edge-weights (black line), imply a lower confidence in the accuracy of the edge-weight estimates between two specific nodes (Epskamp et al., 2018).
(DOCX)

S1 Data Dataset provided as a .xlsx file. (XLSX)
