# Supplementary material for: Interoception, personality, and internet use: Preliminary insights into their association
Source: PLoS One. 2025 Jul 15;20(7):e0328260. doi: 10.1371/journal.pone.0328260 (PMC12262860; doi:10.1371/journal.pone.0328260)
Supplement: S1 File — S1 Table. Spearman’s correlations between interoceptive awareness and personality traits and internet usage patterns. S2 Table. MAIA, Neo-Pi-R and PIUQ-9, DPIU edge weights obtained in the network analysis matrix.S1 Fig. The accuracy and stability of the network for interoceptive awareness, personality traits, and problematic internet use illustrated through bootstrapped confidence intervals (CIs) of estimated edge weights. In the figure, the x-axis represents the estimated edge-weight coefficients, while the y-axis lists each estimated edge weight in descending order from the highest to the lowest mean bootstrap edge-weight. The red line denotes the sample values, and the black line indicates the mean bootstrapped estimated edge-weights. Larger CIs, depicted by a wider shaded area around the mean bootstrapped estimated edge-weights (black line), imply a lower confidence in the accuracy of the edge-weight estimates between two specific nodes (Epskamp et al., 2018). (DOCX) [file pone.0328260.s001.docx]

## Supplementary material

**Table 1**

*Spearman’s Correlations between Interoceptive Awareness and Personality Traits and Internet Usage Patterns.*

| Variable | |  | Noticing | Not-Distracting | Not-Worrying | Attention Regulation | Emotional Awareness | Self-Regulation | Body Listening | Trusting |
| --- | --- | --- | --- | --- | --- | --- | --- | --- | --- | --- |
|  | Personality Traits (Neo-Pi-R) | | | | | | | | | |
| Neuroticism | | Spearman's rho | -0.06 | -0.15 | -0.39* | -0.37* | 0.17* | -0.39 | -0.11 | -0.45* |
|  | | p-value | 0.49 | 0.06 | < .001 | < .001 | 0.03 | < .001 | 0.19 | < .001 |
|  | | n | 156 | 156 | 156 | 156 | 148 | 148 | 148 | 148 |
| Extraversion | | Spearman's rho | 0.05 | 0.08 | 0.08 | 0.06 | 0.19* | 0.17* | 0.19* | 0.20* |
|  | | p-value | 0.57 | 0.35 | 0.32 | 0.47 | 0.02 | 0.04 | 0.02 | 0.02 |
|  | | n | 157 | 157 | 157 | 157 | 149 | 149 | 149 | 149 |
| Openness | | Spearman's rho | 0.20* | 0.08 | -0.05 | 0.13 | 0.28* | 0.18* | 0.28* | 0.05 |
|  | | p-value | 0.01 | 0.32 | 0.51 | 0.11 | < .001 | 0.03 | < .001 | 0.58 |
|  | | n | 156 | 156 | 156 | 156 | 148 | 148 | 148 | 148 |
| Agreeableness | | Spearman's rho | -0.03 | 0.10 | -0.07 | -0.02 | 0.07 | 0.08 | 0.11 | 0.08 |
|  | | p-value | 0.72 | 0.23 | 0.41 | 0.78 | 0.43 | 0.33 | 0.19 | 0.34 |
|  | | n | 157 | 157 | 157 | 157 | 149 | 149 | 149 | 149 |
| Conscientiousness | | Spearman's rho | 0.08 | 0.06 | -0.14 | 0.15 | 0.04 | 0.14 | 0.12 | 0.21* |
|  | | p-value | 0.29 | 0.46 | 0.09 | 0.07 | 0.63 | 0.10 | 0.14 | 0.01 |
|  | | n | 158 | 158 | 158 | 158 | 150 | 150 | 150 | 150 |
|  | Internet Usage Patterns (PIUQ-9, DPIU) | | | | | | | | | |
| PIUQ-9 | | Spearman's rho | 0.03 | -0.36* | -0.08 | 0.06 | 0.10 | -0.04 | 0.06 | -0.16* |
|  | | p-value | 0.73 | < .001 | 0.30 | 0.44 | 0.21 | 0.66 | 0.44 | 0.05 |
|  | | n | 159 | 159 | 159 | 159 | 151 | 151 | 151 | 151 |
| DPIU_total | | Spearman's rho | 0.08 | -0.20* | 0.00 | -0.11 | 0.07 | -0.08 | 0.01 | -0.17 |
|  | | p-value | 0.32 | 0.02 | 0.96 | 0.19 | 0.40 | 0.35 | 0.93 | 0.05 |
|  | | n | 144 | 144 | 144 | 144 | 137 | 137 | 137 | 137 |
| Entertainment and Video Streaming | | Spearman's rho | 0.09 | -0.19 | -0.08 | -0.15 | -0.01 | -0.02 | 0.01 | -0.16 |
|  |  | p-value | 0.37 | 0.06 | 0.43 | 0.13 | 0.95 | 0.82 | 0.90 | 0.13 |
|  |  | n | 102 | 102 | 102 | 102 | 96 | 96 | 96 | 96 |
| Social Media | | Spearman's rho | -0.08 | -0.04 | -0.07 | -0.07 | 0.06 | 0.01 | 0.01 | -0.03 |
|  | | p-value | 0.43 | 0.68 | 0.52 | 0.50 | 0.60 | 0.91 | 0.92 | 0.75 |
|  | | n | 98 | 98 | 98 | 98 | 92 | 92 | 92 | 92 |
| Gaming | | Spearman's rho | 0.16 | -0.09 | -0.23 | 0.12 | 0.24 | 0.28 | 0.31 | 0.12 |
|  | | p-value | 0.35 | 0.62 | 0.18 | 0.50 | 0.16 | 0.10 | 0.07 | 0.48 |
|  | | n | 37 | 37 | 37 | 37 | 36 | 36 | 36 | 36 |
| Messaging | | Spearman's rho | -0.10 | 0.02 | -0.08 | -0.40* | -0.40* | -0.46* | -0.47* | -0.31* |
|  | | p-value | 0.52 | 0.88 | 0.62 | 0.01 | 0.01 | 0.00 | 0.00 | 0.05 |
|  | | n | 43 | 43 | 43 | 43 | 40 | 40 | 40 | 40 |
| Information Search | | Spearman's rho | -0.13 | -0.23 | 0.44* | -0.11 | -0.18 | -0.05 | -0.34 | 0.08 |
|  | | p-value | 0.53 | 0.28 | 0.03 | 0.61 | 0.44 | 0.84 | 0.14 | 0.73 |
|  | | n | 25 | 25 | 25 | 25 | 20 | 20 | 20 | 20 |
| *Note. Significant correlations are marked by asterisks* (*)*. DPIU = Dimensions of Problematic Internet Use; MAIA = Multidimensional Assessment of Interoceptive Awareness; NEO-PI-R = Revised NEO Personality Inventory; PIUQ-9 = Problematic Internet Use Questionnaire.* | | | | | | | | | | |

**Table 2**

*MAIA, Neo-Pi-R and PIUQ-9, DPIU edge weights obtained in the network analysis matrix.*

| **Number of nodes** | **Number of non-zero edges** | | | | | **Sparsity** | | | |  |  |  |  |  |  |  |  |  |
| --- | --- | --- | --- | --- | --- | --- | --- | --- | --- | --- | --- | --- | --- | --- | --- | --- | --- | --- |
| 15 | 33 / 105 | | | | | 0.686 | | | |  |  |  |  |  |  |  |  |  |
| **Variable** | | **Not-Worrying** | **Attention Regulation** | **Emotional Awareness** | **Self-Regulation** | | **Body Listening** | **Trusting** | **Noticing** | | **Not-Distracting** | **Neuroticism** | **Extraversion** | **Openness** | **Agreeableness** | **Conscientiousness** | **PIUQ9** | **DPIU_total** |
| Interoceptive Awareness (MAIA) | | | | | | | | | | | | | | | | | | |
| Not-Worrying | | 0 | 0.112 | -0.117 | 0 | | 0 | 0 | 0 | | 0 | -0.202 | 0 | 0 | 0 | 0 | 0 | 0 |
| Attention Regulation | | 0.112 | 0 | 0 | 0.321 | | 0.079 | 0.095 | 0.161 | | 0 | -0.041 | 0 | 0 | 0 | 0 | 0 | 0 |
| Emotional Awareness | | -0.117 | 0 | 0 | 0 | | 0.445 | 0 | 0.152 | | 0 | 0.018 | 0 | 0.034 | 0 | 0 | 0 | 0 |
| Self-Regulation | | 0 | 0.321 | 0 | 0 | | 0.282 | 0.175 | 0 | | 0 | -0.124 | 0 | 0.04 | 0 | 0 | 0 | 0 |
| Body Listening | | 0 | 0.079 | 0.445 | 0.282 | | 0 | 0 | 0.091 | | -0.025 | 0 | 0 | 0.061 | 0 | 0 | 0 | 0 |
| Trusting | | 0 | 0.095 | 0 | 0.175 | | 0 | 0 | 0 | | 0 | -0.239 | 0.03 | 0 | 0 | 0.063 | -0.04 | -0.011 |
| Noticing | | 0 | 0.161 | 0.152 | 0 | | 0.091 | 0 | 0 | | 0 | 0 | 0 | 0 | 0 | 0 | 0 | 0 |
| Not-Distracting | | 0 | 0 | 0 | 0 | | -0.025 | 0 | 0 | | 0 | 0 | 0 | 0 | 0 | 0 | -0.192 | 0 |
| Personality Traits (Neo-Pi-R) | | | | | | | | | | | | | | | | | | |
| Neuroticism | | -0.202 | -0.041 | 0.018 | -0.124 | | 0 | -0.239 | 0 | | 0 | 0 | -0.029 | 0 | 0 | -0.081 | 0.053 | 0.129 |
| Extraversion | | 0 | 0 | 0 | 0 | | 0 | 0.03 | 0 | | 0 | -0.029 | 0 | 0.267 | 0 | 0 | 0 | 0 |
| Openness | | 0 | 0 | 0.034 | 0.04 | | 0.061 | 0 | 0 | | 0 | 0 | 0.267 | 0 | 0.033 | 0 | 0 | 0 |
| Agreeableness | | 0 | 0 | 0 | 0 | | 0 | 0 | 0 | | 0 | 0 | 0 | 0.033 | 0 | 0 | 0 | 0 |
| Conscientiousness | | 0 | 0 | 0 | 0 | | 0 | 0.063 | 0 | | 0 | -0.081 | 0 | 0 | 0 | 0 | 0 | -0.042 |
| Internet Usage Patterns (PIUQ-9, DPIU) | | | | | | | | | | | | | | | | | | |
| PIUQ9 | | 0 | 0 | 0 | 0 | | 0 | -0.04 | 0 | | -0.192 | 0.053 | 0 | 0 | 0 | 0 | 0 | 0.421 |
| DPIU_total | | 0 | 0 | 0 | 0 | | 0 | -0.011 | 0 | | 0 | 0.129 | 0 | 0 | 0 | -0.042 | 0.421 | 0 |
| *Note. CBOCI = Clark-Beck Obsessive-Compulsive Inventory; DPIU = Dimensions of Problematic Internet Use; MAIA = Multidimensional Assessment of Interoceptive Awareness; NEO-PI-R = Revised NEO Personality Inventory; PIUQ-9 = Problematic Internet Use Questionnaire.* | | | | | | | | | | | | | | | | | | |


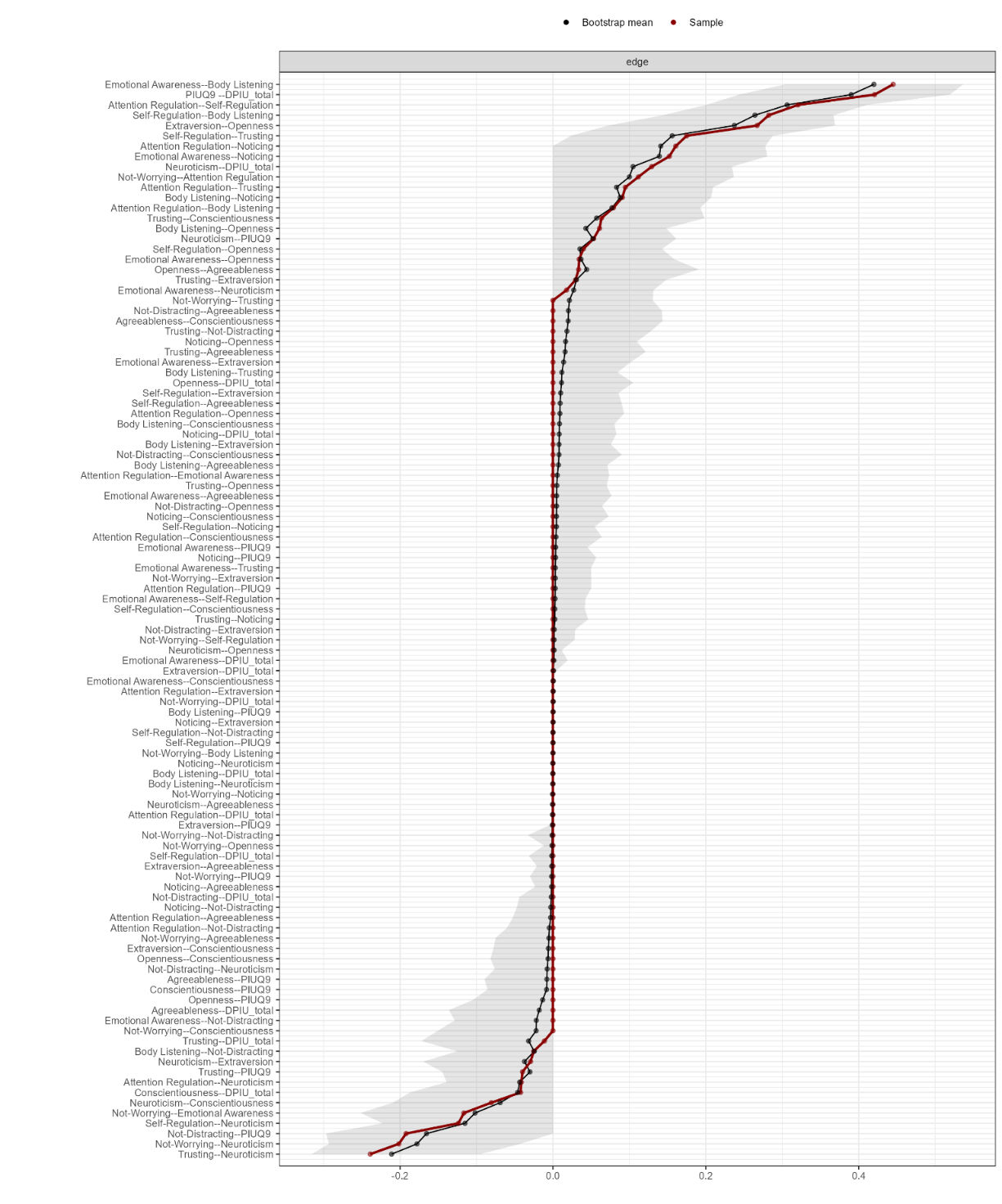


**Figure 1. The accuracy and stability of the network for Interoceptive awareness, Personality traits, and Problematic Internet Use illustrated through bootstrapped confidence intervals (CIs) of estimated edge weights.** In the figure, the x-axis represents the estimated edge-weight coefficients, while the y-axis lists each estimated edge weight in descending order from the highest to the lowest mean bootstrap edge-weight. The red line denotes the sample values, and the black line indicates the mean bootstrapped estimated edge-weights. Larger CIs, depicted by a wider shaded area around the mean bootstrapped estimated edge-weights (black line), imply a lower confidence in the accuracy of the edge-weight estimates between two specific nodes (Epskamp et al., 2018).
